# Garrè Sclerosing Osteomyelitis of the Clavicle: Clinical Results after Clavicular Resection

**DOI:** 10.3390/healthcare12020202

**Published:** 2024-01-15

**Authors:** Andrea Gabriele Calamita, Davide Stimolo, Serena Puccini, Matteo Innocenti, Domenico Andrea Campanacci

**Affiliations:** Department of Orthopaedic Oncology, Careggi University Hospital, 50134 Firenze, Italy; davist93@gmail.com (D.S.); matteo.innocenti@unifi.it (M.I.); domenicoandrea.campanacci@unifi.it (D.A.C.)

**Keywords:** Garrè, sclerosing osteomyelitis, clavicular resection, chronic non-bacterial osteomyelitis

## Abstract

(1) Background: Chronic non-bacterial osteomyelitis (CNO), also known as sclerosing osteomyelitis of Garrè, is a rare inflammatory bone disease with a specific clinical picture, uncertain pathogenesis, and no consensus on an effective treatment. Most frequently affecting other long bones, CNO may rarely involve the clavicle. The aim of this study was to present the results of a series of patients affected by CNO of the clavicle treated with total and partial clavicula resection. In addition, a literature review of different types of treatment of CNO was performed. (2) Methods: We retrospectively reviewed three patients with Sclerosing Osteomyelitis of Garre’ of the clavicle treated with partial resection of the clavicle (one) and with total clavicular resection (two). (3) Results: Patients (two female and one male) were an average age of 35.7 years at the time of the operation. At the 4-year follow-up, the mean active ROM was: 143° forward flexion, 133° abduction, 42° external rotation with an internal rotation of two patients at the interscapular level and one patient at the lumbosacral junction. The mean ASES score was 92/100 (range 87–100). In the literature review, after screening the abstracts and full texts for eligibility, 34 studies met the inclusion criteria. Conclusions: Partial or total clavicular resection resulted an effective treatment of CNO of the clavicle. The procedure seems to be particularly indicated after the failure of more conservative treatments.

## 1. Introduction

Sclerosing osteomyelitis or Chronic non-bacterial osteomyelitis (CNO) is an inflammatory bone disorder, described by Garré in 1893, with typical imaging features, characterized by thickening of the cortices, and loss of the medullary canal without signs of infection [1,2,3].

CNO is a rare disease, more common in children [4] and adolescents, but it can occur in all age groups. The clinical spectrum is variable, ranging from single asymptomatic bone lesions up to the most severe form of chronic recurrent multifocal osteomyelitis (CRMO), a term first used by Björkstén et al. in 1978 [5].

The exact pathogenic mechanisms remain unknown. An infectious origin is excluded. A genetic component in CNO/CRMO has been proposed but never documented in humans, although a strong correlation between CNO/CRMO and personal or familiar history of inflammatory disorders suggests a genetic component. The literature shows that immune dysregulation, particularly of IL-10 and IL-1, may play a role in the etiology of this disease [5].

It can affect any bone, but In children, it occurs most commonly in the metaphyses of the long bones. In adults, the lesion may affect the axial skeleton, with a predilection for the medial end of the clavicles, sternum, and first ribs, which led to the coining of the term sternocostoclavicular hyperostosis (SCCH) for the disorder. Other frequent locations are the spine, the sacrum, the mandible, and the diaphysis of long bones, followed by the pelvis and shoulder girdle [6]

The natural history of CNO is characterized by episodes of exacerbations and remissions of symptoms, followed by a chronic state associated with potentially debilitating pain, irreversible structural changes in affected bones, secondary arthritis, and functional limitations of adjacent joints.

These physical impairments are often associated with psychological sequelae, which may significantly impact various aspects of a patient’s quality of life [7]. The main identified determinants of impaired QoL in adult CNO/SCCH were delay in diagnosis, chronic pain of moderate to high severity, and restricted shoulder girdle function [8].

Other authors have proposed diagnostic criteria and biomarkers for CNO, but these have not been validated in prospective studies, and, to date, CNO/CRMO remains a diagnosis of exclusion [9,10].

Regarding the treatment of CNO in one of the most frequent locations, the clavicular region (about 5% of cases [11]), the literature reports different treatment options. Observation, medical therapy with antibiotics, non-steroidal anti-inflammatory drugs (NSAIDs) or bisphosphonates, TNF inhibitors, disease-modifying antirheumatic drugs (DMARDS), [10,12,13,14,15,16] and surgical excision of the lesion. The surgical treatment for CNO of the clavicle remains debated among intralesional curettage, partial resection, and total clavicular resection (TCR) [17,18,19].

The aim of this study was to analyze the clinical and functional results of a series of patients affected by CNO of the clavicle treated with clavicular resection. In addition, a literature review of different types of treatment of CNO was performed.

## 2. Materials and Methods

This study is a retrospective case series, evaluating CNO of the clavicle surgically treated with total e partial clavicular resection at our Center.

### 2.1. Inclusion Criteria

Were the following: clinical, radiological, and histological diagnosis of CNO, clavicular localization, negative microbiologic cultures, monostotic disease, chronic symptoms, failure of conservative treatment or previous surgeries, and definitive surgical treatment with clavicular resection. 

### 2.2. Exclusion Criteria

Multifocal disease, no clavicular localization, mechanical and neurological pathologies of the upper limb.

### 2.3. Diagnosis

We performed diagnoses in accordance with Jansson et al. [11].

In this work, diagnostic criteria divided into the major and minor have been established.

Major: 1. Radiologically proven osteolytic/-sclerotic bone lesion, 2. Multifocal bone lesions 3. Palmoplantar pustulosis or psoriasis 4. Sterile bone biopsy with signs of inflammation and/or fibrosis.

Minor: A. Normal blood count and good general state of health, B. CRP and ESR mildly to moderately elevated, C. Observation time longer than 6 months, D. Hyperostosis, E. Associated with other autoimmune diseases apart from PalmoPlantarPustolosis (PPP) or psoriasis, F. Grade I or II relatives with autoimmune or autoinflammatory disease or with NBO.

We confirmed the diagnosis with the presence of 2 major criteria or 1 major and 3 minor criteria [11].

Laboratory investigations included assessment of the white blood cell (WBC) count, erythrocyte sedimentation rate (ESR), C-reactive protein (CRP) level, and bacteriologic culture of the biopsy sample.

X-ray, CT scan, and MRI were used for imaging evaluation. Histological diagnosis was made by a pathologist dedicated to musculoskeletal diseases and rare bone diseases. We analyzed early and late postoperative complications requiring medical therapy or reoperation.

### 2.4. Post-Operative Protocol

For the first month, a universal shoulder brace, wrist and elbow mobilization, and passive mobilization up to 90° were used. For the second month, active assisted mobilization was used, and at 6 weeks, muscle strengthening exercises, first isometric then isotonic, and global postural re-education (RPG) were used.

### 2.5. Follow Up

The clinical follow-up, to evaluate the shoulder function and patients’ satisfaction, consisted of the assessment of shoulder range of motion (ROM), American Shoulder and Elbow Surgeons Score (ASES) [20], and Subjective Shoulder Score (SSV) [21].

These assessments were performed at 1, 3, 6, and 12 months in the first year, then with annual checks. 

Imaging follow-up was performed annually with MRI for five years from index surgery.

### 2.6. Surgical Technique

The patient is positioned in supine decubitus under general anesthesia. Draping includes the neck, hemithorax of the affected side, and full arm. Exposure follows the clavicle profile. The Deltoid, Pectoralis Major, and Trapezius muscles are detached from the clavicle. The acromioclavicular joint must be exposed and disarticulated after a section of conoid and trapezoid ligaments. 

**Figure 1:**
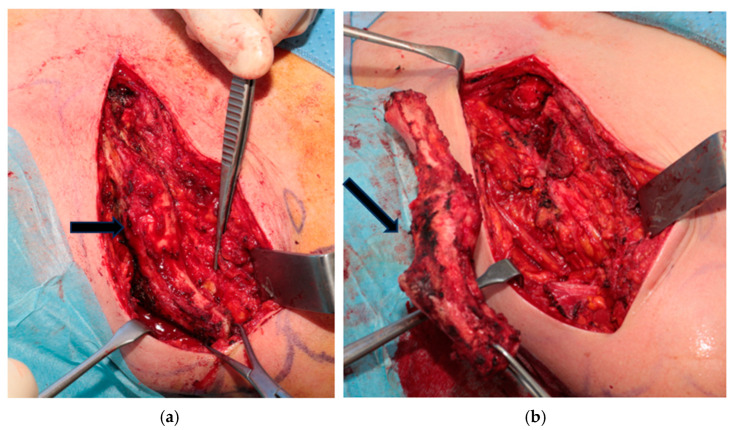
(**a**,**b**) Intraoperative images of total clavicular resection. The arrows indicate the clavicle.

After that, the lateral third of the clavicle is lifted and dissection is continued medially leaving the subclavius muscle to protect the subclavian vascular bundle. Medially, the sternocleidomastoid muscle, the sternoclavicular, and costoclavicular ligaments are sectioned and the sternoclavicular joint is disarticulated, completing the TCR. At the end of the procedure, care must be taken to reconstruct the muscle layer by suturing the trapezius with deltoid and pectoralis major. Then, the fascial plane is closed with an end-to-end re-adsorbable suture, and superficial planes are sutured in layers. (Figure 1) In case of partial clavicular resection, the lateral third of the clavicle can be left in place preserving the insertion of coraco-clavicular ligaments. 

### 2.7. Review

A narrative review of the literature was conducted with a PubMed search for articles between January 2001 and July 2023. Search terms included “Diffuse sclerosing osteomyelitis”, “Chronic sclerosing ostemyelitis of Garrè”, “New treatment option for sclerosing osteomyelitis of Garré”, “management of sclerosing osteomyelitis”, ”Chronic nonbacterial osteomyelitis and chronic recurrent multifocal osteomyelitis”, and “Treatment of chronic recurrent multifocal osteomyelitis”. Furthermore, the identified articles were also cross-referenced.

Three reviewers (M.I, D.S, A.G.C) independently reviewed all titles and/or abstracts and selected studies identified for inclusion. Disagreements were resolved by consensus between the 3 reviewers or by the main author (D.A.C) when required.

Literature reviews, studies not written in English, French, or Spanish, and studies judged to be non-contributory were overlooked.

Relevant studies can be accessed for full-text review to obtain information on age of disease onset, disease location, type of primary treatment performed, follow-up time, treatment outcome, and presence of relapses.

## 3. Results

### 3.1. Case Series

We observed 3 cases of clavicular CNO in 2 females and 1 male. The average age at operation was 35.3 years (19, 30 and 57 years, respectively).

The medial two-thirds of the clavicle were involved in 2 cases, and the whole clavicle in one case.

Patient 1 reported the appearance of symptoms such as local swelling and pain since 2003, patient number 2 since 2005, and patient number 3 since 2016.

The diagnosis occurred, respectively after 15 years from the onset of symptoms for Patient 1, after 13 years for Patient 2, and after 1 year for Patient 3 with an average delay of 9.6 years. 

Patients 1 and 3 had no comorbidities and had never performed upper limb surgery, patient 2 had surgery performed for grade I chondrosarcoma of the ipsilateral proximal humerus. 

All of them had been treated conservatively with NSAIDs, and one patient with additional bisphosphonate therapy and intralesional curettage of the lesion in another hospital. They did not report the use of antibiotics.

In the laboratory investigations, the WBC count was normal in all cases, while ESR and CRP levels were elevated in all cases.

On radiologic evaluation, bone enlargement, osteosclerosis, and spotted lytic areas were present in all cases. 

Histological diagnosis of CNO was obtained with CT-guided core needle biopsy in two patients; in the other one, there was histological diagnosis from excised material of the former curettage. All the cultures were negative for infection.

Patients 1,2 were treated with total clavicular resection, and patient 3 was treated with partial clavicula resection.

Histological examination of the specimens of the 3 patients revealed fibrosclerosis of the medullary spaces, irregular thickening of the bone trabeculae, and no evident neoplastic proliferation.

There were no postoperative complications requiring medical or surgical treatment at early and late follow-up. 

The annual MRI showed no signs of local recurrence and preserved muscle trophism.

At the latest follow-up, no osteoarthritic change in the shoulder or any kyphosis secondary to clavicle resection was observed in any patient.

At the final follow-up, all the patients were free from pain and reported good functional results and no limitations in the use of the affected shoulder during daily living activities. The mean active (a) ROM was: 168.3° forward flexion (aFF), 166.6° abduction (aABD), 41.6° external rotation (aER), and internal rotation (aIR) of two patients at the interscapular level and of one patient at the lumbosacral junction. The individual values are reported in Table 1.

The mean ASES Score to evaluate the function of the shoulder was 92/100 (range 87–100). The mean SSV score showing the patient’s subjective assessment of shoulder function expressed as a percentage of a normal shoulder was 90 (range 85–95).

### 3.2. Literature Review

**Figure 2:**
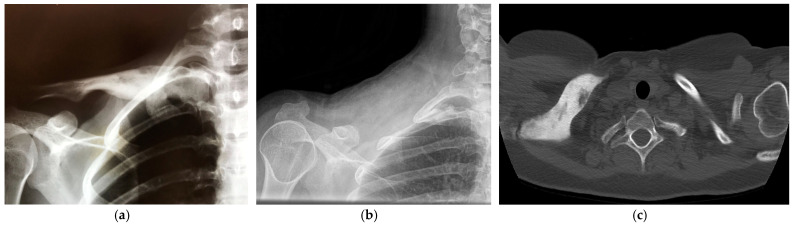
(**a**) X-ray and CT scan (**b**) showing diffuse involvement of the clavicle of patient 1 (**a**) and patient 2 (**b**) with bone thickening and sclerosis including the sternoclavicular joint. (**c**) X-ray after clavicular resection.

**Figure 3:**
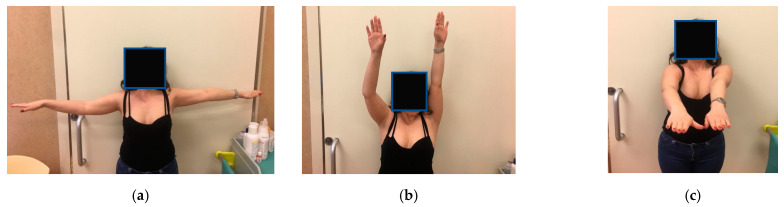
(**a**–**c**) Function at first month follow up after right TCR.

**Figure 4:**
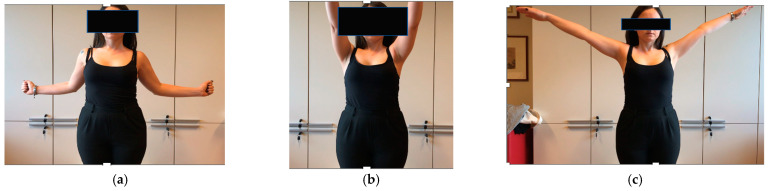
(**a**–**c**) Functional result after right TCR at 4 years follow up.

Our search identified 149 studies potentially focused on the management of chronic non-bacterial osteomyelitis. After screening abstracts and full texts for eligibility, 34 studies met the inclusion criteria (Table 2, Figure 2, Figure 3 and Figure 4).

## 4. Discussion

Chronic non-bacterial osteomyelitis (CNO) is a rare disease [5]. Patients with CNO usually present with chronic pain and progressive swelling over a long period of time.

Ramautar et al. show that adult patients with CNO/SCCH have significant impairment in almost all aspects of QoL, maladaptive illness perceptions, and ineffective coping strategies, compared to healthy and chronically diseased reference populations.

Symptoms and imaging are not specific, and diagnostic workout to exclude other pathologies such as infections or bone tumors is mandatory. After biopsy, histologic diagnosis is usually inconclusive, and the bacterial culture’s result was negative. In most of the cases, when they get appropriate consultation for CNO, patients have already been treated conservatively with antibiotics and NSAIDs. This first-line conservative treatment can be resolutive or can attenuate symptoms for a variable amount of time [22,33,36]. Other studies suggested that bisphosphonates can contribute to pain relief and remission of the disease [26,30,35].

In Table 2, the most recent studies on the treatment of CNO in different sites are reported. From these series, most cases of conservative treatment gave only temporary clinical improvement for the majority of cases. In most of the studies [22,29,36,39,48,49,51], medical therapy consisted of different combinations of NSAIDs, antibiotics, and corticosteroids.

In the last years, other studies [43,44,45,46,47,50,53,54] proved the efficacy of TNF inhibitors and human monoclonal antibodies targeting IL-1 as a treatment after the failure of NSAIDs, corticosteroids, and bisphosphonates. Their use was frequently associated with symptom relief and long remission intervals of disease, especially in children with multiple localizations. Even low-dose radiotherapy was reported successful in one study [42].

Despite the improvement of symptoms in some cases, the efficacy of conservative treatment with medical therapy remains unpredictable, resulting in failure and persistence of the disease in many cases.

On the contrary, several studies report clinical improvement [25,30,41] or resolution [23,27,28,31,32,33,37,38,40] of symptoms after surgical treatment with debridement [25,31,32,38,41], surgical resection [27,40] alone, or combined with reconstruction [23,28,30,33,37] in the tibial or femoral site.

Intralesional curettage represents the first choice, but it is not always successful for definitive resolution of symptoms [25,41]. Thus, in selected cases, it is necessary to consider more radical options such as, in the setting of clavicle CNO, a partial or total clavicle resection.

In the literature, only one article focused in detail on a case of clavicular CNO [34] reporting on a 12-year-old girl with Garrè osteomyelitis localized at the medial segment. Three cycles of antibiotics (fusidic acid and cloxacillin) were administered without complete resolution of symptoms. At the final follow-up, after 5 years of conservative treatment, persistent medial clavicle swelling with recurrent pain was present. A recent systematic review [55] analyzed the results of treatment of 294 clavicle osteomyelitis, 146 bacterial, and 148 non-bacterial, reporting a healing rate of 90% after nonsurgical treatment and 80% after surgical treatment, with no distinction between infective and non-infective osteomyelitis.

The clavicle plays multiple functions: to protect the underlying neurovascular structures, to provide an attachment for musculotendinous structures, and to connect the upper limb to the trunk [56]. Maintaining the appropriate length and tension of the periscapular muscles is important in order to optimize the biomechanical lever arm and to improve ROM, strength, and functional efficacy of the shoulder girdle [57,58]. However, excellent shoulder stability and function can be recovered even after TCR, with an accurate muscular plane reconstruction and appropriate specific muscular reinforcement. Indeed, Rubright et al. [59] evaluated 5 patients after TCR comparing strength, ROM, kinematic analysis, and functional tests between the affected shoulder and the contralateral side. Even though the objective measure of strength and ROM was reduced in the limb without clavicle and kinematic analysis showed scapular dyskinesia and a significant reduction in external rotation, there was no difference in DASH and SF-36 scores and patients had a normal perception of their health and quality of life.

Total clavicular resection was first mentioned in 1912 and later described in detail by Gurd [60]. Indications for total clavicle resection can be tumors, infections, pseudarthroses, and chronic pain after trauma [61,62]. A recent review of the literature by Nistor et al. highlights that claviculectomy, with or without reconstruction, is the elective indication for malignant tumors of the clavicle [63].

Several studies in the literature reported good results after TCR. Oheim [64] et al., reported 5 patients treated with total clavicle resection for osteitis. The average Constant score showed a significant improvement, from 82 to 95, after surgery. Also, Krishnan [65] et al. described improvement in the ASES score in a report including 6 patients receiving TCR.

The indication for TCR might lead to different outcomes: Wessel and Schaap [66] reported good results after TCR for patients with chronic osteitis and malignancy but unsatisfying results in patients with chronic post-traumatic pain, even if there were no differences considering the range of movement. Other studies confirmed good results after TCR in oncological patients [67,68]. The reconstruction of the clavicle after resection does not seem to be necessary and advisable. Li [68] et al. reported a higher Costant–Murley score and faster functional recovery in patients who underwent TCR with no clavicle reconstruction than in patients with allograft replacement.

In our series, TCR proved to be effective in curing CNO, and no case of disease recurrence or pain resumption was observed. Despite the abnormal kinematics, patients’ perceptions of upper extremity global function and their overall quality of life remained satisfactory and did not deteriorate with time.

## 5. Conclusions

Clavicular resection allows excellent functional results by providing definitive healing from CNO with a clear improvement in various aspects of the patient’s quality of life.

It should be proposed to patients suffering from CNO after failure of the other lines of treatment.

Garre osteomyelitis represents a rare pathological condition making it challenging to formulate a study with an adequate dataset to achieve statistically significant results.

In the future, multicenter studies could provide a larger cohort of patients useful to confirm and strengthen current data regarding clavicular resection in CNO.

Furthermore, our results confirm the difficulty in making a correct and early diagnosis of this pathology.

We think that greater knowledge of the topic and entrusting the management of these patients to specialist centers can improve the diagnosis of this rare disease.

## Figures and Tables

**Table 1:** Patient characteristics and postoperative function.

	Patient 1	Patient 2	Patient 3
**Age**	30	57	19
**Sex**	F	F	M
**Side** **Dominat art**	Right yes	Left no	Right yes
**Preop treatment**	NSAID, bisphosphonate, currettage	NSAIDs	NSAIDs
**Postoperative aFF**	170°	165°	170°
**Postoperative aABD**	170°	160°	170°
**Postoperative aER**	50°	30°	45°
**Postoperative aIR**	Interscapular level	Interscapular level	Lumbosacral junction
**Follow-up (years)**	5	5	6
**ASES score**	100	87	89.9
**SSV (%)**	95	85	90

**Table 2:** Review of the literature.

NO	Year	Author	Age/Sex	Site	Primary Therapy	Follow-Up	Outcome
**1**	2001	Segev, E. [22]	3 pts	Tibia	NSAID + antibiotics	45–114 months	1 Improved 2 Failure
**2**	2002	Klisch, J. [23]	15/M	Calvarium	Surgical resection + reconstruction	2 years	Cure
**3**	2002	Belli, E. [24]	17/F	Mandible	Antibiotics + oxygen therapy	NA	Failure
**4**	2003	Sharma, H. [25]	20/F	Metatarsal	Curettage	1 years	Improved
**5**	2004	Wright, S.A. [26]	21/F	Femur	Risedronate	NA	Improved
**6**	2005	Viejo-Fuertes, D. [27]	14/M	Fibula	Surgical resection	6 months	Cure
**7**	2005	Kelkar, A.S. [28]	15/F	Metacarpal	Surgical resection + reconstruction + antibiotics	1 years	Cure
**8**	2006	Nasir, N. [29]	40/M	Sacrum	Antibiotics	16 months	Improved
**9**	2006	Armstrong, D.J. [30]	27/F	2 pts	Ibandronate	NA	1 Improved 1 Failure
**9**	2006	Armstrong, D.J. [30]	38/F	Tibia + Femur	Surgical resection + reconstruction + intramedullary nailing + ibandronate	NA	Improved
**10**	2007	Suma, R. [31]	10/M	Mandible	Surgical curettage + antibiotics	3 years	Cure
**11**	2008	Tavera, A.R. [32]	13/M	Femur	Debridement surgery + antibiotics	NA	Cure
**12**	2010	Schwartz, A.J. [33]	11/M	Humerus	Surgical resection + vascularized fibular autograft + NSAID	35 months	Cure
**13**	2012	Pan, K. [34]	12/F	Clavicle	Antibiotics	5 years	Failure
**14**	2012	Urade, M. [35]	61/F	Mandible	Pamidronate	15 years	Improved
**15**	2013	Franco-Jiménez, S. [36]	6/F	Sacrum	Corticosteroid + NSAID	2 years	Improved
**16**	2013	Nikomarov, D. [37]	15/F	Tibia	Surgical resection + reconstruction	30 months	Cure
**17**	2014	Mooney, J.F. [38]	7/M	Femur	Debridement surgery + antibiotics	NA	Cure
**18**	2014	Elera-Fitzcarrald, C. [39]	35/M	Femur	Antibiotics	4 months	Improved
**19**	2014	Vannet, N.B. [40]	50/F	Femur	Intramedullary nailing + antibiotics	8 years	Cure
**20**	2014	de Moraes, F.B. [41]	54/F	Tibia + femur	Surgical curettage + drainage + antibiotics	NA	Improved
**21**	2017	Dietzel, C.T. [42]	67/F	Tibia + Talus	Low dose radiotherapy	10 months	Cure
**22**	2017	Moussa, T. [43]	7 pts	Multiple	TNF inhibitor (infliximab—canakinumab)	1.5 years	Improved
**23**	2017	Schnabel, A. [44]	55 pts	Multiple	NSAIDs + corticosteroids + TNF inhibitor	5 years	Improved
**24**	2018	Tronconi, E. [45]	4 pts	Multiple	NSAIDs + corticosteroids + TNF inhibitor	14 months	Improved
**25**	2018	Ramraj, R. [46]	16/M	Vertebra (extraintestinal manifestation of IBD)	TNF inhibitor	1 years	Cure
**26**	2019	Kostik, M.M. [47]	52 pts	Multiple	NSAIDs vs. Pamidronate vs. TNF inhibitor vs. MTX	NA	Improved
**27**	2019	Andreansen, C.M. [48]	51 pts	Multiple	Pamidronate	4 years	Improved
**28**	2019	Sulko, J. [49]	41 pts	Foot, hip, clavicle	Pamidronate	21 months	32 Cure 9 Improved
**29**	2020	Bustamante, J. [50]	19 pts	Multiple	TNF inhibitor	67 months	10 Improved 9 Cure
**30**	2020	Yang, X. [51]	5/F	Jaw	Alendronate + vitamin D3	1 years	Cure
**31**	2021	Barbur, I. [52]	Case report	Jaw	Splint	2 years	Cure
**32**	2022	Georgaki, M. [53]	6.5/F	Jaw	Etanercept, corticosteroids and methotrexate	1 years	Cure
**33**	2022	Acierno [54]	13/F	Multiple	28(Canakinumab)	1.8 years	Improved

NSAID: non-steroidal anti-inflammatory drugs; TNF: Tumor necrosis factor; MTX: methotrexate; Cure: resolution of symptoms; IMPROVED: improvement of symptoms without resolution; FAILURE: no improvement in symptoms.

## Data Availability

Data are contained within the article.
